# Metagenomics of gut microbiome for migratory seagulls in Kunming city revealed the potential public risk to human health

**DOI:** 10.1186/s12864-023-09379-1

**Published:** 2023-05-19

**Authors:** Feng Liao, Jing Qian, Ruian Yang, Wenpeng Gu, Rufang Li, Tingting Yang, Xiaoqing Fu, Bing Yuan, Yunhui Zhang

**Affiliations:** 1 Department of Respiratory Medicine, The First People’s Hospital of Yunnan Province, 650022 Kunming, P.R. China; 2 The Affiliated Hospital of Kunming University of Science and Technology, 650500 Kunming, P.R. China; 3 Department of Acute Infectious Diseases Control and Prevention, Yunnan Provincial Centre for Disease Control and Prevention, 650022 Kunming, P.R. China

**Keywords:** Gut microbiome, Migratory seagulls, DNA virome, RNA virome

## Abstract

**Background:**

Seagull as a migratory wild bird has become most popular species in southwest China since 1980s. Previously, we analyzed the gut microbiota and intestinal pathogenic bacteria configuration for this species by using 16S rRNA sequencing and culture methods. To continue in-depth research on the gut microbiome of migratory seagulls, the metagenomics, DNA virome and RNA virome were both investigated for their gut microbial communities of abundance and diversity in this study.

**Results:**

The metagenomics results showed 99.72% of total species was bacteria, followed by viruses, fungi, archaea and eukaryota. In particular, *Shigella sonnei*, *Escherichia albertii*, *Klebsiella pneumonia*, *Salmonella enterica* and *Shigella flexneri* were the top distributed taxa at species level. PCoA, NMDS, and statistics indicated some drug resistant genes, such as *adeL*, *evgS*, *tetA*, *PmrF*, and *evgA* accumulated as time went by from November to January of the next year, and most of these genes were antibiotic efflux. DNA virome composition demonstrated that Caudovirales was the most abundance virus, followed by Cirlivirales, Geplafuvirales, Petitvirales and Piccovirales. Most of these phages corresponded to *Enterobacteriaceae* and *Campylobacteriaceae* bacterial hosts respectively. *Caliciviridae*, *Coronaviridae* and *Picornaviridae* were the top distributed RNA virome at family level of this migratory animal. Phylogenetic analysis indicated the sequences of contigs of *Gammacoronavirus* and *Deltacoronavirus* had highly similarity with some *coronavirus* references.

**Conclusions:**

In general, the characteristics of gut microbiome of migratory seagulls were closely related to human activities, and multiomics still revealed the potential public risk to human health.

**Supplementary Information:**

The online version contains supplementary material available at 10.1186/s12864-023-09379-1.

## Background

China is one of the countries with the most abundant animal resources in the world [1]. As the important biological resources, wild animals play the pivotal role in maintaining ecological balance and biodiversity [2]. The possibility of animal migrations to disseminate pathogens across different geographic areas has brought about several studies on the interactions between migration and infection [2–4]. Migratory birds can carry pathogens on their migrations, especially those that do not significantly affect their health. The research on infectious diseases of wild birds started relatively late in China. After the world’s first large-scale highly pathogenic avian influenza outbreak in Qinghai Lake in 2005, much more attention has been paid for investigate the migratory wild birds [5–7].

Seagull (*Larus ridibundus*) as a migratory wild bird has become most popular species in southwest China since 1980s. Each year, large numbers of seagulls migrate from Siberia to Southern China to overwinter, and many citizens come to feed them on parks or rivers. Thus, the public are in close contact with migratory seagulls. Previously, we analyzed the gut microbiota and intestinal pathogenic bacteria configuration for this species by using 16S rRNA sequencing and culture methods respectively [8]. Although there was little cross-infection between humans and seagulls in previous study, many pathogenic bacteria were isolated from feces of this animal, such as enteropathogenic *Escherichia coli*, *Salmonella* spp. and *Yersinia* spp. [8]. However, the amplicon sequencing and culture-based studies only reflect limited findings of microbial communities of animals. Now, advanced methods have been used in-depth analysis on the connection between bird activities and disease dissemination, particularly, the metagenomics and meta-transcriptomics provide us the broader characterization of microbe diversity, including bacteria, virus and fungi [9–11]. In this study, the metagenomics, DNA virome and RNA virome of migratory seagulls were both investigated for their gut microbiome of abundance and diversity.

## Results

### Metagenomics

The average effect reads of total samples was 99.74% ± 0.05%, and Q30 value after quality control was 89.83% ± 1.36%. 0.28% ± 0.052% of host reads was filtered. The average assembled contigs was 58,350 ± 33,306 of total samples, and predicted gene numbers was 68,462 ± 28,605.

The KEGG pathway results indicated that 103,321 genes annotated in metabolism, followed by 19,361 in environmental information processing, 10,820 in cellular processes, 8,334 in genetic information processing, 8,182 in human diseases and 3,161 in organismal systems, as Additional file 1a shown. The heatmap of top 25 pathways revealed highly relative abundance of genes distributed in HG group, especially for HG-3 and HG-4 (Additional file 1b). The eggNOG function classification showed cell wall/membrane/envelope biogenesis, transcription, amino acid transport and metabolism were the top distributed genes (Additional file 1c). The CARD annotation demonstrated that *macB*, *bcrA*, *OmpK37*, *msbA*, *tetA(58)*, *evgS*, *AbaF*, *cpxA*, *oleC* and *adeL* were the top 10 relative abundance of antibiotic resistant genes. Most of these genes belonged to antibiotic efflux, as Additional file 1d shown. The top 10 distributed virulence factors between HG and HXQ groups were different (Additional file 1e). Polar-flagella, BfmRS and CdpA had highly relative abundance in HG group, compared to Capsule, AcrAB and Enterobactin in HXQ group. The PHI annotation results revealed reduce virulence (1,494), unaffected pathogenicity (732) and increased virulence (244) were the top three phenotype classifications, as Additional file 1f shown.

The composition of gut microbiome indicated that 99.72% of total species was bacteria, followed by viruses (0.2%), fungi (0.07%), archaea (0.001%) and eukaryota (0.0002%). Proteobacteria (95.7%) and Firmicutes (4%) were the most distributed abundance at phylum level of bacteria, and Gammaproteobacteria (Class level) accounted for 99.7% of Proteobacteria. The relative abundance of each sample at different taxonomic levels of bacteria was shown in Additional file 2. In general, Proteobacteria, Gammaproteobacteria, Enterobacterales, *Enterobacteriaceae*, *Escherichia* and *Escherichiacoli* were the most distributed abundance of each sample at different taxonomic levels, except HG-4 sample. In particular, *Shigella sonnei*, *Escherichia albertii*, *Klebsiella pneumonia*, *Salmonella enterica* and *Shigella flexneri* were the top distributed relative abundance at species level, and all these bacteria were considered as human pathogens (Additional file 2).

**Fig. 1:**
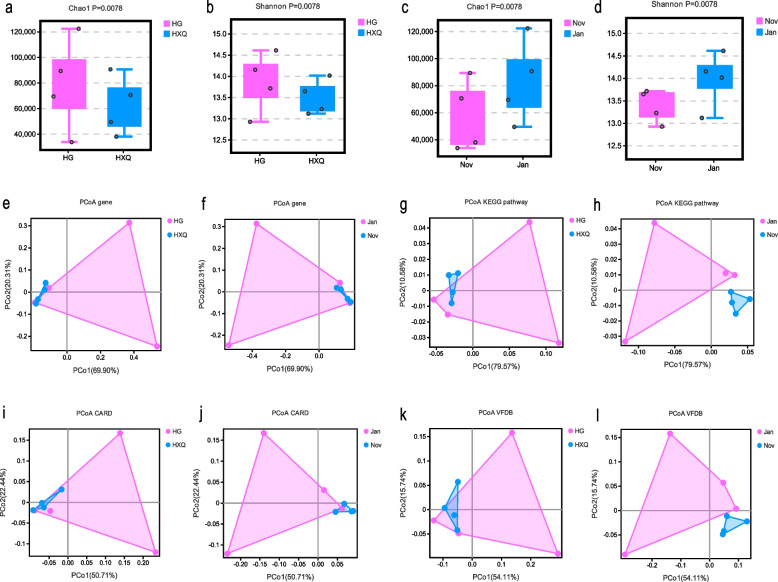
Alpha and Beta diversity of metagenomic of seagulls’ feces. **a** The Chao1 index between HG and HXQ group. **b** The Shannon index between HG and HXQ group. **c** The Chao1 index between Nov and Jan group. **d** The Shannon index between Nov and Jan group. **e** The PCoA of annotated genes between HG and HXQ group. **f** The PCoA of annotated genes between Nov and Jan group. **g** The PCoA of KEGG pathways between HG and HXQ group. **h** The PCoA of KEGG pathways between Nov and Jan group. **i** The PCoA of annotated CARD between HG and HXQ group. **j** The PCoA of annotated CARD between Nov and Jan group. **k** The PCoA of annotated VFDB between HG and HXQ group. **l** The PCoA of annotated VFDB between Nov and Jan group

Alpha diversity showed the Chao1, Ace, Shannon and Simpson index of samples were 70,348.40 ± 28,041.63, 70,217.06 ± 28,062.91, 13.67 ± 0.54 and 0.99 ± 0.0005 respectively. Chao1 and Shannon index showed the statistical significance (*P* = 0.0078) between HG and HXQ groups (Fig. 1a and b), and significant differences were also found between Nov and Jan groups for two alpha diversity index (Fig. 1c and d). Beta diversity indicated that no statistical significance were found both for species composition of sample location groups (HG vs HXQ, *P* = 0.297) and time groups (Nov vs Jan, *P* = 0.356), as Fig. 1e and f shown. PCoA of KEGG pathway showed that two clusters were generated according to location and time groups respectively (Fig. 1g and h), but Anosim only revealed the statistical difference between HG and HXQ group (*P* = 0.028). PCoA of CARD showed the similar clustering results with KEGG pathway (Fig. 1i and j), and statistical significance was found between Nov and Jan group (*P* = 0.02). Anosim analysis demonstrated no differences were found both for location and time groups of VFDB diversity, although independent clustering of each group was identified (Fig. 1k and l).

**Fig. 2:**
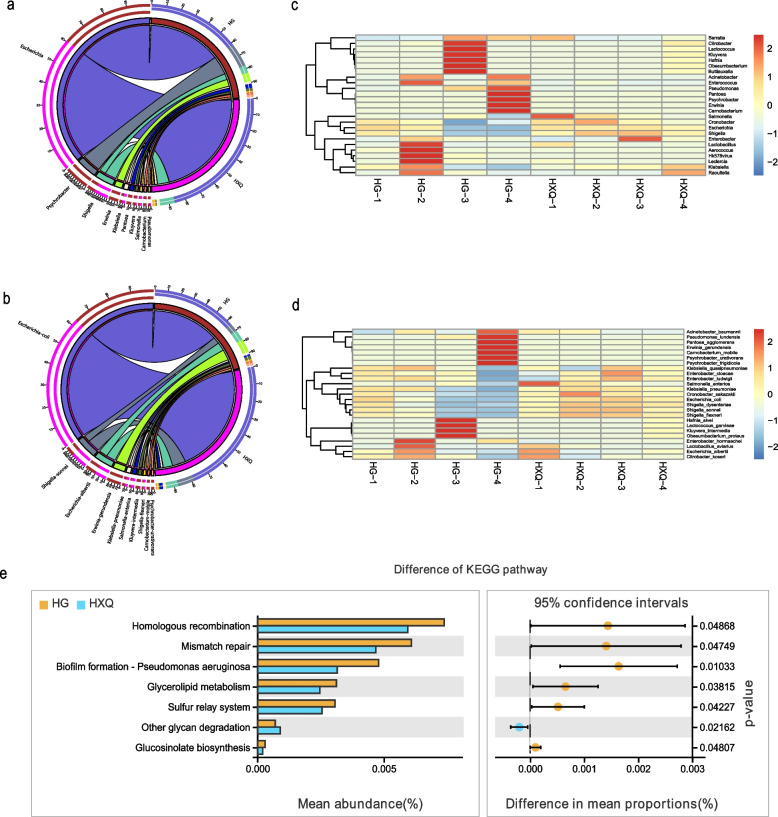
Comparison the difference of species composition between HG and HXQ group. **a** The circos diagram of species composition at genus level between HG and HXQ group. **b** The circos diagram of species composition at species level between HG and HXQ group. **c**. The heatmap of distribution for top 25 of species composition in each group at genus level. **d** The heatmap of distribution for top 25 of species composition in each group at species level. **e** The statistical significance of KEGG pathway using Welch’s t test between HG and HXQ group

**Fig. 3:**
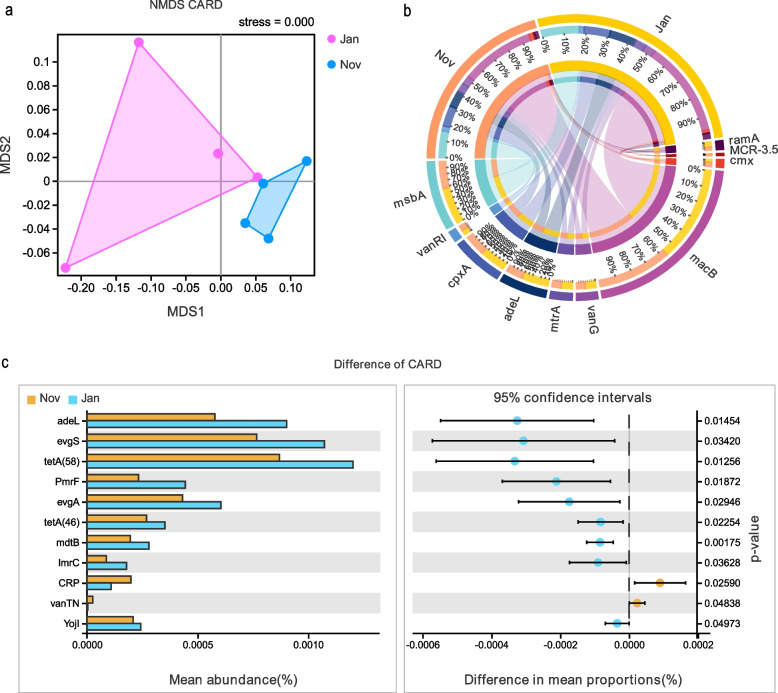
Beta diversity and statistics of CARD annotations between Nov and Jan group. **a** The NMDS of CARD annotation results between Nov and Jan group. **b** The circos diagram of top 10 relative abundance of annotated CARD genes between Nov and Jan group. **c** The statistical significance of CARD annotated genes using Welch’s t test between Nov and Jan group

Difference analysis indicated that highly species composition was found in HG group compared to HXQ group. At genus level, *Escherichia*, *Psychrobacter*, *Shigella*, *Erwinia*, *Kluyvera*, *Pantoea*, *Carnobacterium* and *Pseudomonas* were identified in HG group (Fig. 2a). At species level, *E. coli*, *S. sonnei*, *E. albertii*, *E. gerundensis*, *K. pneumonia*, *S. enterica*, *K. intermedia*, *C. mobile* and *P. urativorans* had highly relative abundance in HG group in accordance with genus level (Fig. 2b). The distribution of top 25 of species composition in each group was different as well. The highly diversity was found in HG group (from HG-1 to HG-4), but *S. enterica*, *K. pneumonia*, *S. dysenteriae*,* S. sonnei* and *S. flexneri* had highly relative abundance in HXQ group (from HXQ-1 to HXQ-4), as Fig. 2c and d shown. Welch’s t test of KEGG pathway showed homologous recombination, mismatch repair, biofilm formation, glycerolipid metabolism and sulfur relay system had highly relative abundance in HG group (Fig. 2e). In addition, the NMDS of CARD between Nov and Jan group showed two independent clusters, and Anosim indicated the statistical significance (R = 0.333, *P* = 0.02), as Fig. 3a shown. The circos diagram demonstrated the top 10 relative abundance of CARD annotated genes between Nov and Jan group; *macB*, *msbA*, *cpxA*, *adeL*, *mtrA* and *vanG* genes had highly abundance in Jan group (Fig. 3b). Welch’s t test revealed *adeL*, *evgS*, *tetA*, *PmrF*, *evgA*, *mdtB*, *lmrC* and *YojI* genes had higher abundance in Jan group compared to Nov group (Fig. 3c). Most of these genes were antibiotic efflux, and resistant to fluoroquinolone, macrolide, tetracycline and peptide antibiotics.

### DNA virome

The average effect reads of total samples was 76.03% ± 8.41% for DNA virome, and 99.89% ± 0.23% of clean reads was obtained after host reads filtration. The average assembled contigs was 64,856 ± 38,055 of total samples. Virus identification and classification results showed 78.30% (152,724/195,046) of contigs was identified as phage, 1.15% (2,248/195,046) was other virus, and 20.55% (40,074/195,046) was unassigned (Additional file 3a). The dsDNA virus accounted for 77.38% (150,930/195,046), ssDNA virus accounted for 1.72% (3,353/195,046), and 20.90% (40,761/195,046) was unassigned (Additional file 3b). UniProt functional annotation results indicated 37,436 genes annotated for technical term, 25,421 for molecular function, and 24,609 for biological process, as Additional file 3c shown. KEGG annotations at three levels revealed most of genes belonged to metabolism, genetic information processing, signaling and cellular processes, DNA replication proteins, DNA repair and recombination proteins, replication and repair (Additional file 3d to f).

**Fig. 4:**
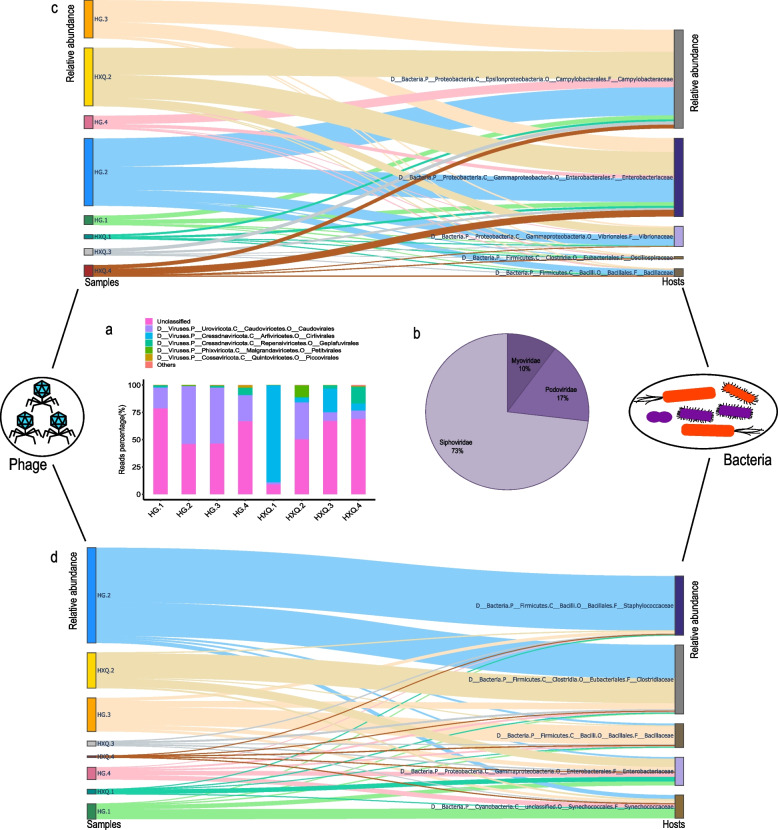
The characteristics of gut DNA virome of migratory seagulls. **a** The histogram of DNA viral composition results at order level for each sample. **b** The constitution of Caudovirales at family level of migratory seagulls for total samples. **c** The phage-host prediction based on Space Pharer. **d** The phage-host prediction based on VPF-Class prediction

Viral composition results at order level demonstrated that Caudovirales (24.81%) was the most abundance DNA virus, followed by Cirlivirales (15.37%), Geplafuvirales (3.47%), Petitvirales (1.6%) and Piccovirales (0.41%). 54.09% of total reads was unclassified (Fig. 4a). *Siphoviridae*(72.52%), *Podoviridae*(16.80%) and *Myoviridae*(9.88%) constituted most of Caudovirales (Fig. 4b) at family level. Phage-host prediction based on Space Pharer showed the top 5 of relative abundance of bacterial hosts were *Campylobacteraceae*, *Enterobacteriaceae*, *Vibrionaceae*, *Oscillospiraceae* and *Bacillaceae* at family level corresponded to each sample (Fig. 4c). *Staphylococcaceae*, *Clostridiaceae*, *Bacillaceae*, *Enterobacteriaceae* and *Synechococcaceae* were the top 5 relative abundance of bacterial hosts according to VPF-Class prediction (Fig. 4d).

Alpha diversity showed the Chao1, Ace, Shannon and Simpson index of samples were 15,335.93 ± 11,516.37, 15,700.62 ± 11,896.91, 8.14 ± 1.96 and 0.96 ± 0.047 respectively. Chao1 and Shannon index showed the statistical significance (*P* = 0.048 and 0.03) between HG and HXQ groups (Additional file 4a and b), but no significant differences found between Nov and Jan groups (Additional file 4c and d). PCoA analysis indicated two clusters were generated according to location and time groups respectively (Additional file 4e and f). However, Anosim only revealed the statistical difference between Nov and Jan group (*P* = 0.027).

LEfSe analysis showed the significantly different abundance between HG and HXQ group at viral order level (Additional file 4 g). The biomarker of HG group was Caudovirales compared with Cirlivirales in HXQ group. The biomarkers of different abundance between Nov and Jan group at viral order level were Geplafuvirales, Piccovirales, Kalamavirales and Rowavirales (Additional file 4 h). In addition, several contigs were also showed significant abundance between Nov and Jan group, as Additional file 4i shown. There were 17 contigs of biomarkers in Nov group compared with 34 contigs in Jan group.

### RNA virome

**Fig. 5:**
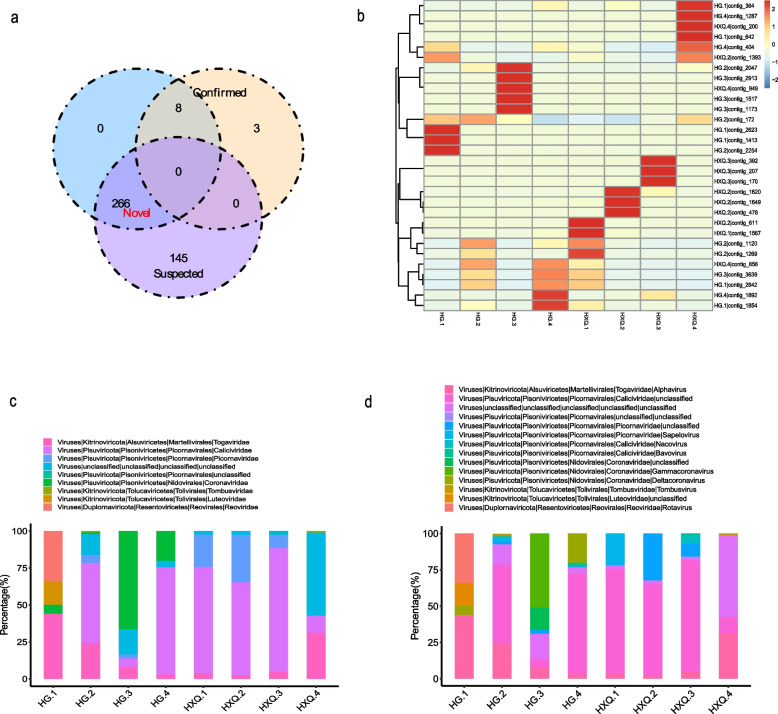
The characteristics of gut RNA virome of migratory seagulls. **a** The venn diagram for comparison of two methods of RNA virome identification. **b** The heatmap of top 30 abundance of RNA viruses contigs in each sample. **c** The top10 abundance of viruses at family level for RNA virome. **d** The top 10 abundance of viruses at genus level for RNA virome

The average effect reads of total samples was 73.52% ± 1.64% for RNA virome, and 71.78% ± 8.58% of clean reads was obtained after host reads filtration. The average assembled contigs was 2,392 ± 898.03 of total samples. Based on reference virus identification method, 11 contigs of RNA viruses were recognized as confirmed, and 411 were considered as suspected. The *Denovo* method showed 274 contigs of RNA viruses were identified as novel. The comparison results of two methods were shown in Fig. 5a, the confirmed and novel contigs of RNA viruses were used for next step analysis. 50.54% (140/277) of contigs were annotated as *Caliciviridae*, followed by *Coronaviridae* (24.55%, 68/277) and *Picornaviridae* (5.42%, 15/277). The heatmap of top 30 abundance of viruses indicated that each sample had the special abundance distribution feature (Fig. 5b), and highly relative abundance of RNA viruses contigs were shown in different samples. *Caliciviridae*, *Picornaviridae*, *Togaviridae*, *Coronaviridae*, *Reoviridae*, *Luteoviridae* and *Tombusviridae* were the top 10 abundance of viruses at family level for RNA virome (Fig. 5c). *Alphavirus*, *Gammacoronavirus*, *Deltacoronavirus*, *Tombusvirus*, *Rotavirus*, *Nacovirus* and *Bavovirus* were the top 10 abundance of viruses at genus level (Fig. 5d).

**Fig. 6:**
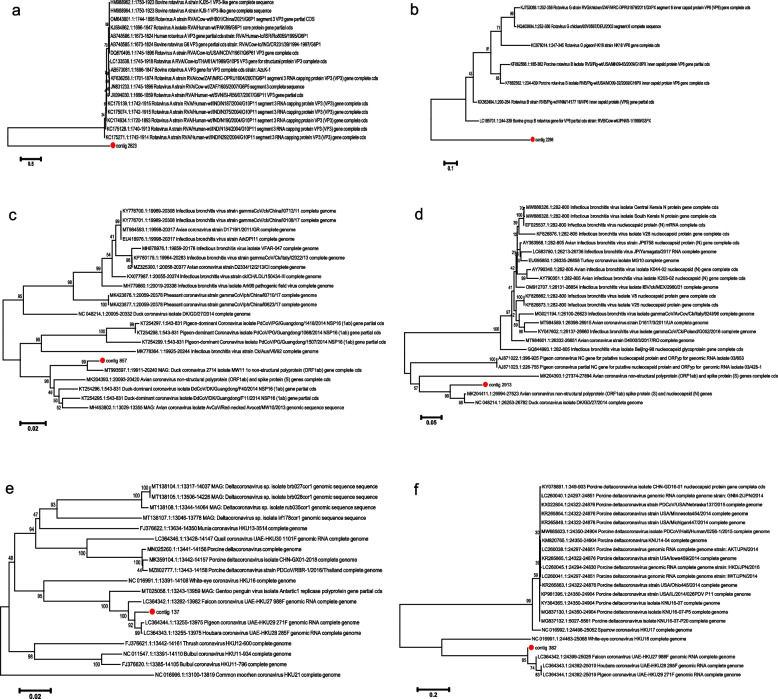
Phylogenetic analysis of some contigs of RNA virome with references. **a** Phylogenetic tree of contig 2623 with *Rotavirus A* references. **b** Phylogenetic tree of contig 2266 with *Rotavirus G* references. **c** Phylogenetic tree of contig 857 with *Gammacoronavirus* references. **d** Phylogenetic tree of contig 2913 with *Gammacoronavirus* references. **e** Phylogenetic tree of contig 137 with *Deltacoronavirus* references. **f** Phylogenetic tree of contig 382 with *Deltacoronavirus* references

Phylogenetic analysis indicated contig 2623 belonged to *Rotavirus A*, showing 71.82% similarity with reference (GenBank accession: KC175271) (Fig. 6a and Additional file 5). Contig 2266 showed the 75% similarity with *Rotavirus G* (GenBank accession: HQ403604.1), as Fig. 6b and Additional file 5 shown. Contig 857 and contig 2913 were both belonged to *Gammacoronavirus*, showing 96.67% and 89.25% similarity with *Duck coronavirus 2714* and *Avian coronavirus* respectively (GenBank accession: MT993597.1 and NC_048214.1) (Fig. 6c, d and Additional file 5). Contig 137 and 382 were *Deltacoronavirus*, showing 97.78% and 96.82% similarity with *Houbara coronavirus UAE-HKU28* and *Pigeon coronavirus UAE-HKU29* (GenBank accession: LC364343.1 and LC364344.1), as Fig. 6e, f and Additional file 5 shown.

### Verification of NGS data

Real-time PCR for *Salmonella* spp. showed positive results for all metagenomic nucleic acid samples, while 20 environmental samples (including 10 soil and 10 water samples) were negative for PCR detection (Additional file 6a). The identical results were found in Additional file 6b for *Shigella* spp. detection.

PCR and amplicon sequencing of contig 2623 in RNA virome showed that the NGS nucleotide sequence of contig 2623 was identical to that of the PCR amplicon by sequence alignment (Additional file 6c). In addition, NGS results for contig 857 and contig 137 in RNA virome were fully consistent with PCR amplicon sequencing results, as Additional file 6d and e shown.

## Discussion

Due to their geographical dispersal ability and wide distribution range, birds not only have strong environmental adaptability, but also bring potential risks to the rapid spread of diseases [12]. Since the twentieth century, bird diseases represented by avian cholera, West Nile fever, and avian influenza have occurred frequently, resulting in the death of numerous wild birds, poultry and even humans [13, 14]. Several studies showed the gut microbiota of waterfowl, using culture-based methods and mainly concerned on some pathogens [15, 16]. Because culture-based studies only provide a limited finding of natural microbial communities, culture-independent molecular methods can be used to describe the composition of waterfowl gut microbiome. Our previous results showed *Enterococcaceae*, *Enterobacteriaceae*, and *Mycoplasmataceae* were the most predominant families of gut microbiota of seagulls by 16S rRNA amplicon sequencing. The most isolated intestinal pathogenic bacteria from seagulls were enteropathogenic *E. coli* and *Salmonella* by culture-based method. The results of the homology analysis of the strains by PFGE method showed that there was little cross-infection between humans and gulls, despite close proximity [8]. In this study, we used metagenomic and meta-transcriptomics techniques to in-depth reveal the features of gut microbiome of migratory seagulls. The results demonstrated that *S. sonnei*, *E. albertii*, *K. pneumonia*, *S. enterica* and *S. flexneri* were the top distributed taxa at species level by metagenomic investigation. All these bacteria were considered as human pathogens, and it was still a potential public risk to human health.

Birds are commonly recognized as key species in dissemination of antimicrobial agents through their feces or polluted water [12, 13, 17]. The problem is that migratory birds can fly over thousands of distances, enabling dissemination across continents. The gulls have been found to be reservoirs of *beta-lactams* resistance in Europe, especially for Spain [18]. In this study, some drug resistance genes accumulated as time went by from November to January of the next year. PCoA, NMDS, and statistics indicated some CARD genes had higher abundance in Jan group compared to Nov group, and most of these genes were antibiotic efflux. This phenomenon probably reflected that when seagulls migrated to Kunming city, the abundance of some drug resistant genes in their entero-intestinal tracts gradually increased. In addition, the diversity of species of HG was higher than that of HXQ. HG is closer to the wild environment, and HXQ is in the human habitation environment, which may be the reason for the difference. In KEGG pathway, the types of HG metabolic pathway genes were significantly higher than those of HXQ. As the taxonomic composition, the heatmaps of species and genus level showed the diversity of HG was significantly higher than the distribution of HXQ. The proportion of *Salmonella*, *Shigella*, *Klebsiella* and other related human pathogens was relatively higher in HXQ group, while highly diversity of species was shown in HG group. From another side, it was also revealed the relative restriction of living environment of seagulls once they settled down in a certain region. From the annotation of drug resistance and distribution of gut microbiome in different locations, the higher abundance of pathogenic bacteria and CARD genes were found in region closer to human living area. However, seagulls lived in wild-like environment had highly biodiversity of gut microbial community, fewer pathogenic bacteria and drug resistant genes.

Bacteriophages can affect composition of microbial communities, abundance and interact with their hosts frequently. Until now, most of the recognized phages are dsDNA, tailed phages belonging to the Caudovirales [19]. For the DNA virome of seagulls, Caudovirales was also the most abundance DNA virus. *Siphoviridae*, *Podoviridae* and *Myoviridae*constituted most of Caudovirales in this study. *Coronavirus* have caused three worldwide epidemics in recent two decades, including the ongoing pandemic of SARS-CoV-2 [20]. Large numbers of animals can serve as reservoirs of *Coronavirus*, including mice, birds and bats [21]. *Alphacoronavirus* and *Betacoronavirus* infect mammals, while *Gammacoronavirus* and *Deltacoronavirus* infect birds. Chu et al. identified three strains of *Deltacoronavirus* in fecal samples of migratory seagulls in Yunnan province [22]. The highly similarity of genomes and amino acid sequence to the homologs of *falcon coronavirus UAE-HKU27*, *houbara coronavirus UAE-HKU28*, and *pigeon coronavirus UAE-HKU29* were both found. They concluded that there might be cross-border transmission of these avian *Deltacoronavirus* through specific routes. Interestingly, in this study we also found *Gammacoronavirus* and *Deltacoronavirus* of gut RNA virome in migratory seagulls. Phylogenetic analysis indicated the sequences of contigs of *Deltacoronavirus* had highly similarity with *Houbara coronavirus UAE-HKU28* and *Pigeon coronavirus UAE-HKU29* as mentioned above. Therefore, *coronaviruses* were possibly ubiquitous in the gut of migratory seagulls.

## Conclusions

In this study, the metagenomics, DNA virome and RNA virome were both used to analyze the gut microbiome of migratory seagulls in southwest China. Some human entero-pathogens were the top distributed taxa by metagenomic investigation, and some antibiotic resistant genes were accumulated as time went by. Bacteriophages were the most abundance of DNA virome corresponded to *Enterobacteriaceae* and *Campylobacteriaceae* bacterial hosts respectively. *Rotavirus*, *Gammacoronavirus* and *Deltacoronavirus* were commonly found in RNA virome in this animal, and *Gammacoronavirus* contigs found in the present study might co-relate with the human *coronavirus* infection. In general, multiomics of gut microbiome for migratory seagulls still revealed the potential public risk to human health.

## Methods

### Sample collection

A total of 160 seagull fecal samples were collected at November 2021 and January 2022. Samples were collected according to two time points (Nov and Jan) and locations (HXQ and HG), and 80 samples were collected from one place at single time point, and the sampling from 2 selected locations was done simultaneously. HXQ is a central park in the urban area, which is closer to the living environment of the human. HG is the surrounding area of Dianchi Lake of Kunming city, which is far from the living environment of the people and closer to the natural environment. The distance between the two sampling locations was approximately 11 km. Apart from the migratory seagull, no other birds of the same species were active and defecating in the above mentioned areas. The rationale for choosing these two sites was mainly based on the proximity of one sampling site to the natural environment and the other to areas of human activity. Every 20 samples of each group were mixed into one for subsequent experimental analysis, and the mixed sample was collected around 5–10 m far from each other for each sampling location or time point.

As soon as the seagull feces were excreted, we collected and stored them in sterile containers immediately to avoid the environmental contamination. Meanwhile, 20 environmental samples included 10 soil and 10 water samples were also collected from the surrounding areas of the seagulls at the same time points.

### Metagenomics

Genomic DNA was extracted using fecal sample DNA extraction kits (Tiangen, Beijing) according to the manufacturer’s instructions. The DNA quality was detected using Qubit 2.0 (Thermo Fisher Scientific) and Nanodrop accordingly. Qualified genomic DNA was fragmented by sonication to a size of 350 bp, and then end-repaired, A-tailed, and adaptor ligated using NEB Next DNA Library Prep Kit for Illumina (NEB, USA) according to the instructions. DNA fragments with length of 300–400 bp were enriched by PCR, and purified using AMPure XP system (Beckman Coulter, USA). The libraries were analyzed for size distribution by 2100 Bio-analyzer (Agilent, CA) and quantified using real-time PCR [23]. Finally, genome sequencing was performed on the Illumina Novaseq 6000 by pair-end 150 (PE150) reagent kit.

Raw data were filtered using FASTP (version 0.18.0). Clean reads of each sample were assembled individually using MEGAHIT (version 1.1.2) stepping over a k-mer range of 21 to 141 to generate sample (or group)-derived assembly [24]. Genes were predicted based on the final assembly contigs (> 500 bp) using MetaGeneMark (version 3.38) [25]. The reads were aligned to predict genes using Bowtie (version 2.2.5) to count reads numbers [26]. Final gene catalogue was obtained from non-redundant genes with gene reads count > 2. The plots were graphed using R ggplot2 package [27]. The unigenes were annotated using DIAMOND (version 0.9.24) by aligning with the deposited ones in different databases including Nr, KEGG, eggNOG, CAZy, PHI, VFDB, and CARD [28–31]. Circular layout representations of functional gene abundance were graphed using circos (version 0.69–3) [32].

Chao1, ACE, Shannon, Simpson index were calculated using Python package (version 0.5.6). Alpha index comparison between groups was calculated by Welch’s t-test and Wilcoxon rank test. Bray–curtis distance matrix based on gene/taxon/function abundance was generated by R Vegan package. Multivariate statistical techniques including PCA, PCoA and NMDS were calculated and plotted using R ggplot2 package. Adonis and Anosim test was calculated using R project Vegan package [33]. Heatmap graph were plotted using R Pheatmap package.

### DNA virome

Two grams of each fecal sample were used and added 5 volumes of pre-cooled sterile SB buffer, vortex for 5 min. Centrifuged the sample at 12,000 g for 5 min to remove the precipitate, removed the cell fragments by 0.45 μm and 0.22 μm filter membrane, and the supernatant was transferred to the ultracentrifugation tube containing 28% sucrose, then centrifuged at 160,000 g for 2 h at 4 °C with HIMAC CP 100 WX ultracentrifuge (Hitachi, Japan). After removing the supernatant, resuspended the pellet in 200 μl SB buffer, added a mixture of DNase I (TaKaRa) and RNase A (TaKaRa) to digest unprotected nucleic acid and incubated at 37 °C for 60 min; added SS solution (2 μl) to inactivate the enzyme reaction at 65–75 °C for 10 min, centrifuged at 2,000 g for 5 min, and stored 200 μl of the supernatant at -20 °C [34].

DNA viruses in samples were extracted by using Magen DNA extraction kits (Magen, China), and the whole genome was amplified with Qiagen kit (REPLI-g Cell WGA & WTA Kit). The DNA quality was detected using Qubit 4.0 (Thermo Fisher Scientific) and 1.5% agarose electrophoresis. Sequencing libraries were generated using NEB Next Ultra DNA Library Prep Kit for Illumina (NEB, USA) following manufacturer’s recommendations. The library was sequenced on an Illumina Novaseq 6000 and 150 bp paired-end reads were generated.

The raw data were filtered using Soapnuke (v1.5.6) to acquire the clean data [35]. BWA software (v0.7.17) was used to compare clean reads with ribosome database (Silva.132) and host database respectively. The clean data were assembled by Megahit software (v1.1.2). The potential viral sequences in the assembled sequence were first predicted using CheckV software [36]. The virus identification was performed again using Virsorter2 software [37]. The distribution of virus reads according to the annotation results of virus contigs were counted by BWA software, and the RPKM of each virus contigs were calculated [38]. Metagenemark (v3.38) was used to predict the gene sequence of the virus contigs and evaluate the predicted gene numbers. The protein sequence of gene was compared with the virus sequence of UniProtKB/Swissprot database (ViralZone, https://viralzone.expasy.org/) by using Blastp software (v2.9.0 +), and the best hit comparison result of e < 1e-3 was screened to obtain the virus function information [39].

The abundance and diversity of DNA viruses were reflected by the analysis of Alpha and Beta Diversity as mentioned above. LDA Effect Size (LEfSe) analyse was used to find the biomarker of each group based on RPKM of virus sequence. CRISPR Recognition Tool (CRT, http://www.room220.com/crt/) was used to construct the CRISPR-CAS spacer database from bacterial genome of RefSeq database. The blastn-short (v2.9.0 +) was used to compare the identified viruses with CRISPR CAS spacer database, the best hit was selected as the possible host information of phage when the e-value < 1e-10, the comparison similarity was more than 95%, and the coverage of spacer was more than 80%.

### RNA virome

The RNA viruses of enrichment of fecal samples were performed as mentioned above. RNA viruses in samples were extracted by using Magen RNA extraction kits (Magen, China), and the whole genome was amplified with Qiagen kit (REPLI-g Cell WGA & WTA Kit).

Sequencing libraries construction and sequencing method were identical as DNA virome. The bioinformatics analyses were similar as mentioned above. Specifically, Blast software (v2.9.0 +) was used to compare the unique contig with virus database, if the alignment similarity was ≥ 80%, the alignment length was ≥ 500 bp, and e ≤ 1e-5 was defined as virus sequence; if the alignment length was ≥ 100 bp, and e ≤ 1e-5 was defined as suspected virus sequence. The candidate viruses sequences were queried against the NCBI reference genomes using BWA-MEM, NT database using MegaBLAST (e-value cutoff 1E-10), BLASTn (e-value cutoff 1E-10), and NCBI NR database using BLASTx (e-value cutoff 1E-3). Sequences with significant hits were classified as novel viruses based on the taxonomy identity of the best BLAST hit [40, 41]. Parts of the viruses’ contigs, such as *Rotavirus*, *Gammacoronavirus* and *Deltacoronavirus* were aligned using Blast software to compare their similarity with other references. MEGA 6.0 software was used to build the phylogenetic tree using N-J methods with 1000 bootstraps.

### Verification of NGS data

Total genome of soil samples were extracted by using soil genomic DNA extraction kit (Tiangen, Beijing) and water samples were concentrated by using 0.45 μm filtration (Sartorius, German), followed by extracted of their genomic DNA. All environmental extracted DNA samples as well as metagenomic DNA library of seagull feces were detected for *Salmonella* and *Shigella* spp. by using real-time PCR kits (Bioperfectus, Jiangsu). The purpose of this experiment was to verify the accuracy of NGS data and rule out the possibility of environmental contamination.

In order to verify the NGS results of the novel viruses in RNA virome, we designed PCR primers for contig 2623, contig 857 and contig 137 (Additional file 7). PCR amplification was performed using 20 μl system, containing 10 μl of Taq premix, 8 μl of water, 0.5 μl of upstream and downstream primers (10 μmol), and 1 μl of cDNA library of extracted RNA. The products were observed by 1.5% agarose gel electrophoresis and sent for bidirectional sequencing to Shuoqing, Kunming. MEGA 6.0 software was used for sequence alignment.

### Availability of data

All data generated or analyzed during this study are included in this published article. The sequence data have been deposited into the National Center for Biotechnology Information (NCBI), https://www.ncbi.nlm.nih.gov/ with BioProject accession number: PRJNA849401 for metagenomics; PRJNA847753 for DNA virome and PRJNA848403 for RNA virome.

## Supplementary Information

**Additional file 1.**

**Additional file 2.**

**Additional file 3.**

**Additional file 4.**

**Additional file 5.**

**Additional file 6.**

**Additional file 7.**

## Data Availability

The datasets generated and/or analysed during the current study are available in the National Center for Biotechnology Information (NCBI), https://www.ncbi.nlm.nih.gov/ with BioProject accession number: PRJNA849401 for metagenomics; PRJNA847753 for DNA virome and PRJNA848403 for RNA virome.

## Abbreviations

PCoA: Principal Co-ordinates Analysis
NMDS: Non-metric multidimensional scaling
KEGG: Kyoto Encyclopedia of Genes and Genomes
CARD: Comprehensive Antibiotic Resistance Database
LEfSe: Linear discriminant analysis Effect Size
