# Supplementary material for: Metagenomics of gut microbiome for migratory seagulls in Kunming city revealed the potential public risk to human health
Source: BMC Genomics. 2023 May 19;24:269. doi: 10.1186/s12864-023-09379-1 (PMC10196292; doi:10.1186/s12864-023-09379-1)
Supplement: Supplementary file 1 — Additional file 1. [file 12864_2023_9379_MOESM1_ESM.pdf]

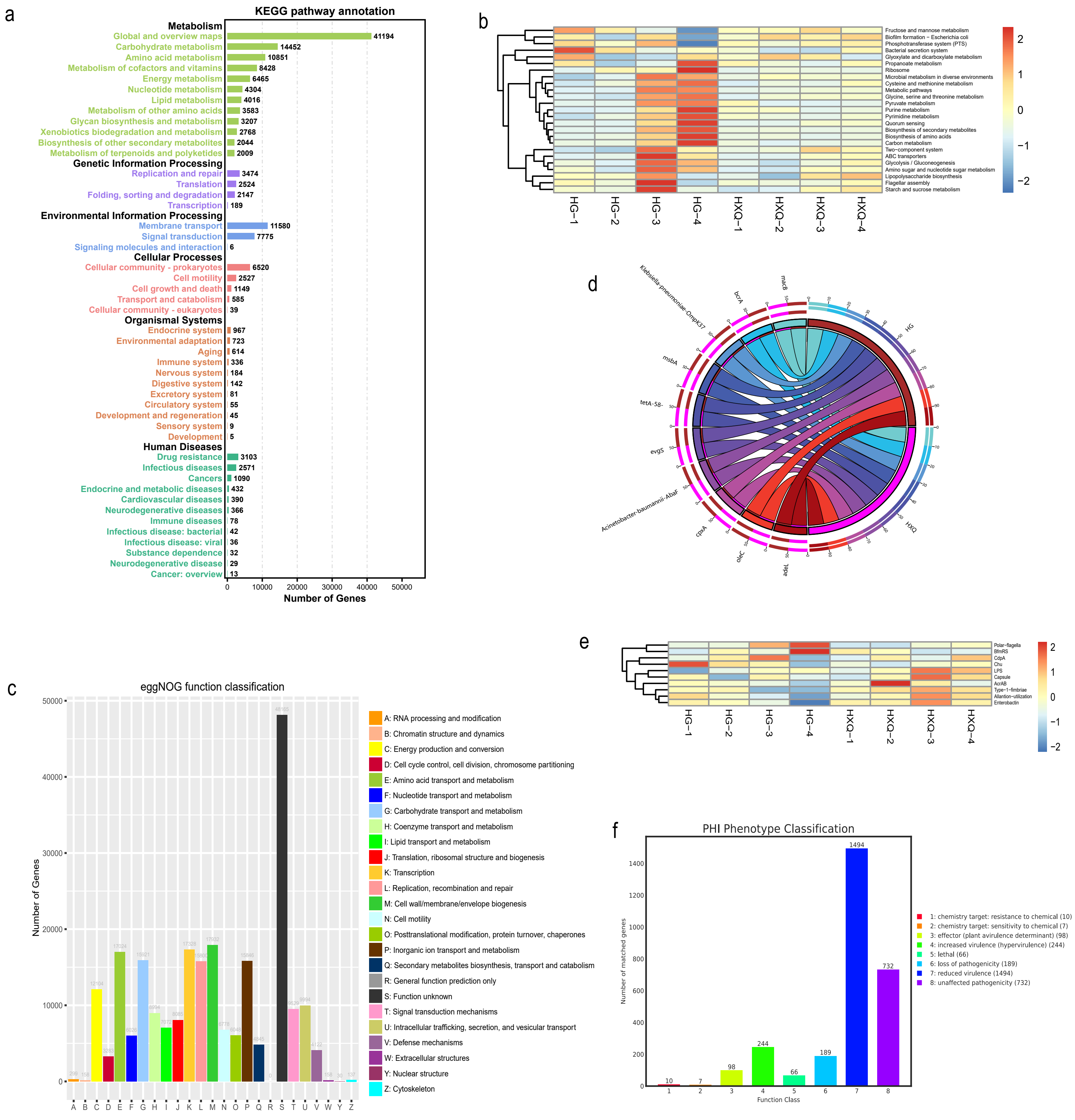

Additional file 1. The annotation results of gut metagenomic of seagulls.

a. The KEGG pathway annotation results

b. The heatmap of top 25 of relative abundance for pathway

c. The eggNOG function classification results

d. The CARD annotation results between HG and HXQ group

e. The top 10 distributed virulence factors between HG and HXQ group

f. The PHI annotation results
