# Supplementary material for: Metagenomics of gut microbiome for migratory seagulls in Kunming city revealed the potential public risk to human health
Source: BMC Genomics. 2023 May 19;24:269. doi: 10.1186/s12864-023-09379-1 (PMC10196292; doi:10.1186/s12864-023-09379-1)
Supplement: Supplementary file 2 — Additional file 2. [file 12864_2023_9379_MOESM2_ESM.pdf]

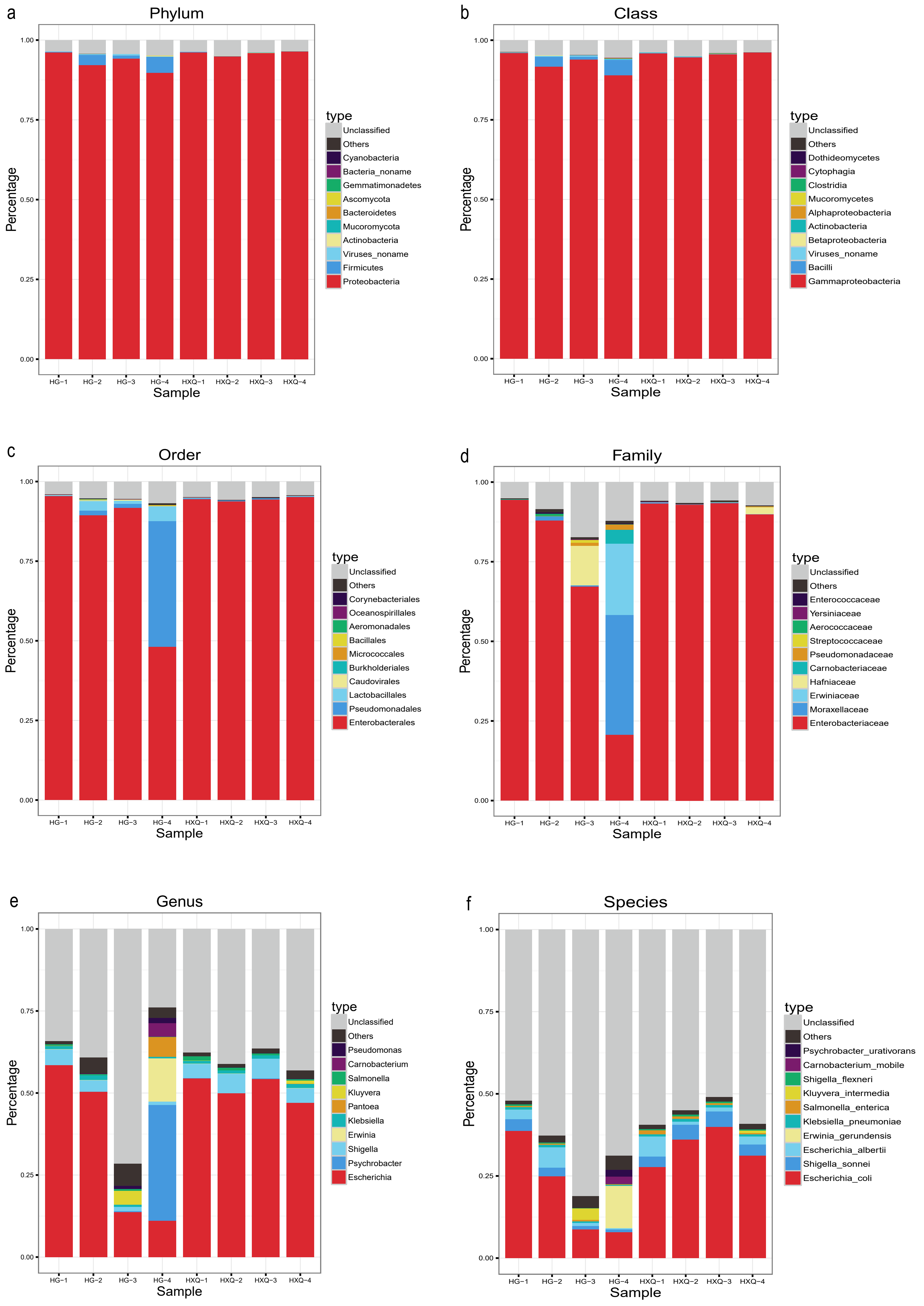

Additional file 2. The relative abundance of each sample at different taxonomic level of bacteria.

a. The relative abundance of each sample at phylum level

b. The relative abundance of each sample at class level

c. The relative abundance of each sample at order level

d. The relative abundance of each sample at family level

e. The relative abundance of each sample at genus level

f. The relative abundance of each sample at species level
