# Supplementary material for: Metagenomics of gut microbiome for migratory seagulls in Kunming city revealed the potential public risk to human health
Source: BMC Genomics. 2023 May 19;24:269. doi: 10.1186/s12864-023-09379-1 (PMC10196292; doi:10.1186/s12864-023-09379-1)
Supplement: Supplementary file 3 — Additional file 3. [file 12864_2023_9379_MOESM3_ESM.pdf]

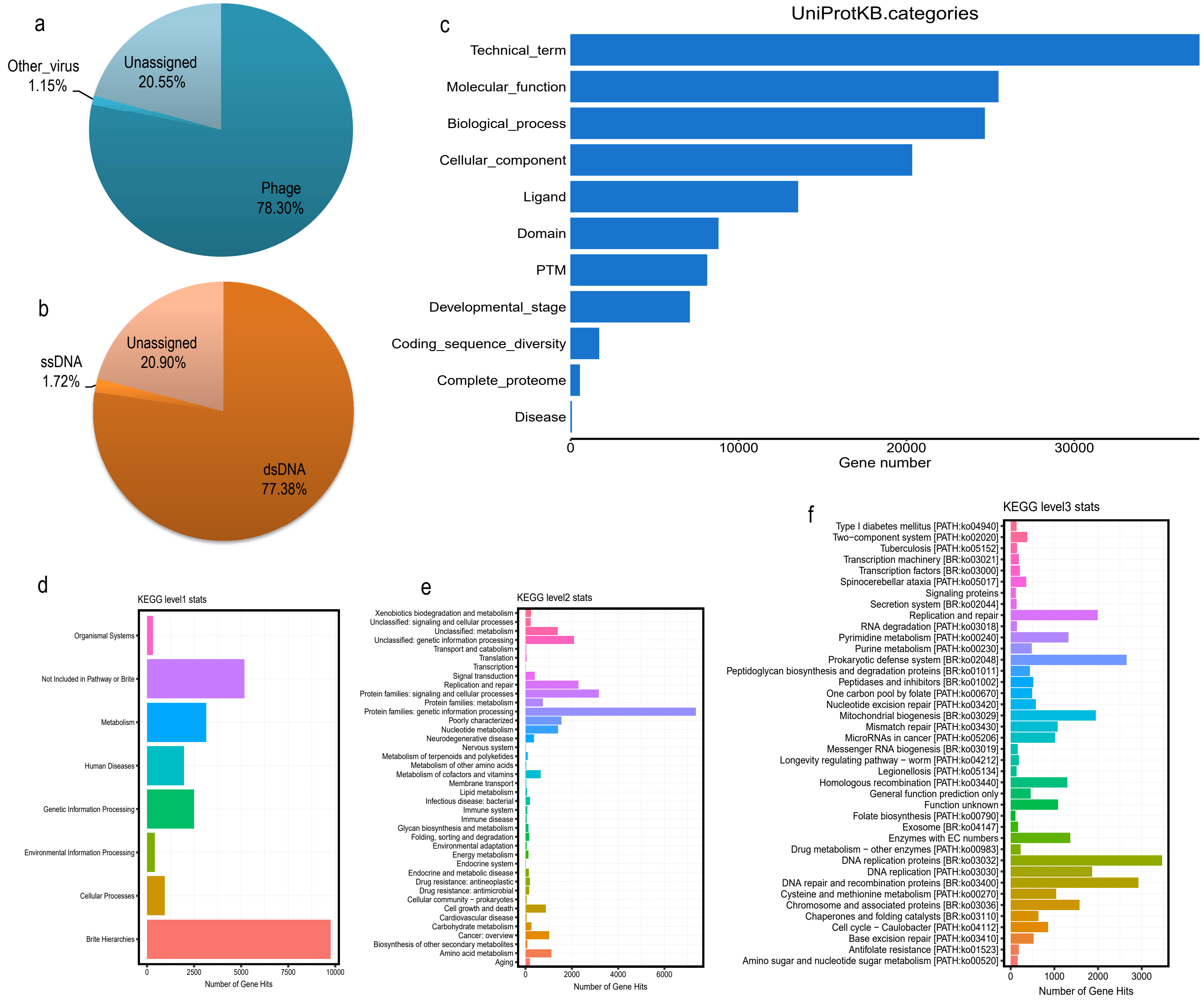

Additional file 3. The annotation results of gut DNA virome of migratory seagulls.
a. Virus identification results of DNA virome
b. Virus classification results of DNA virome
c. The UniProt functional annotation results
d. KEGG annotation results in level 1
e. KEGG annotation results in level 2
f. KEGG annotation results in level 3
