# Supplementary material for: Metagenomics of gut microbiome for migratory seagulls in Kunming city revealed the potential public risk to human health
Source: BMC Genomics. 2023 May 19;24:269. doi: 10.1186/s12864-023-09379-1 (PMC10196292; doi:10.1186/s12864-023-09379-1)
Supplement: Supplementary file 4 — Additional file 4. [file 12864_2023_9379_MOESM4_ESM.pdf]

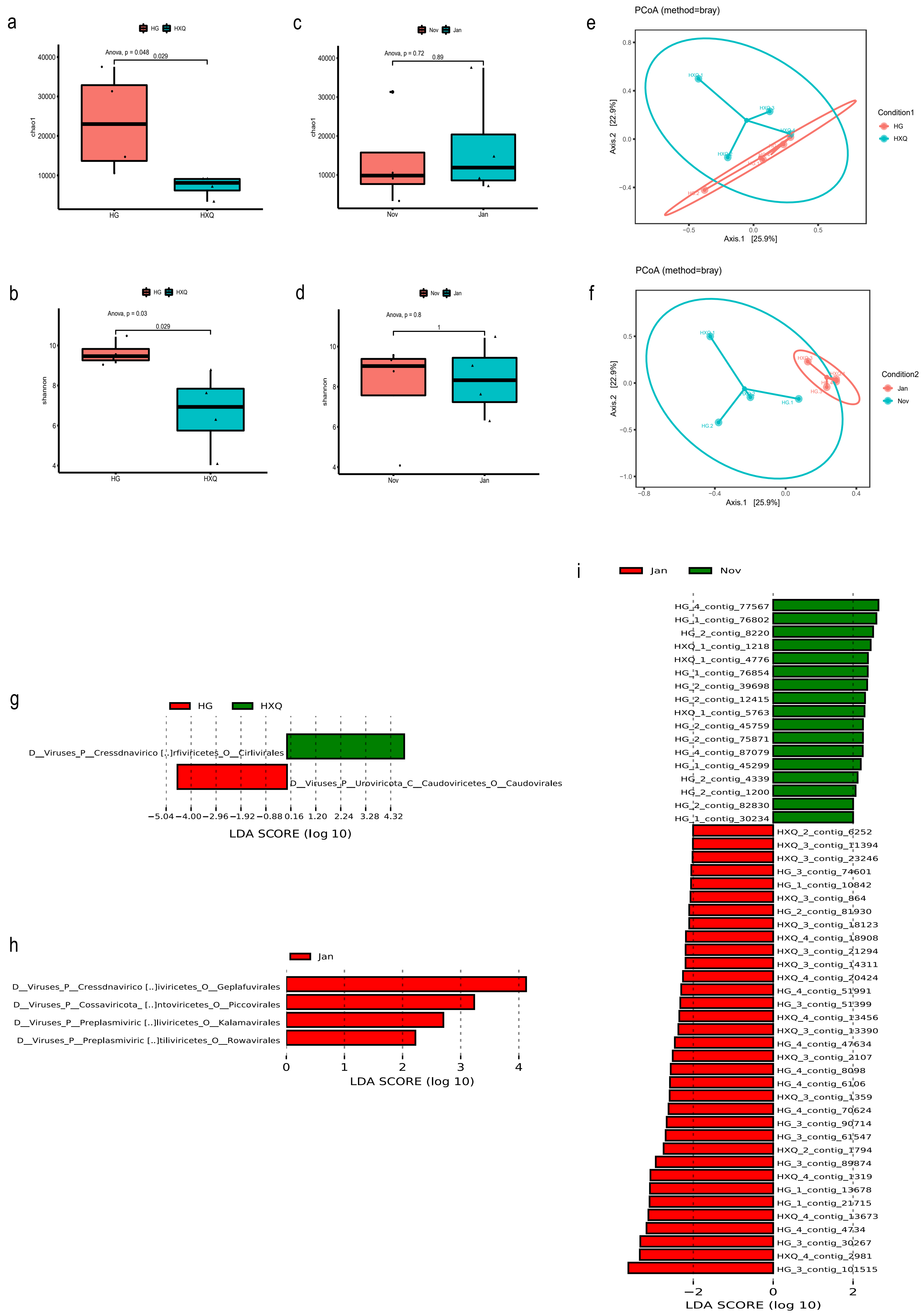

Additional file 4. The diversity of gut DNA virome of migratory seagulls in this study.
a. The Chao1 index between HG and HXQ group
b. The Shannon index between HG and HXQ group
c. The Chao1 index between Nov and Jan group
d. The Shannon index between Nov and Jan group
e. The PCoA of annotated contigs between HG and HXQ group
f. The PCoA of annotated contigs between Nov and Jan group
g. LEfSe analysis of different abundance between HG and HXQ group at order level
h. LEfSe analysis of different abundance between Nov and Jan group at order level
i. LEfSe analysis of different abundance of contigs between Nov and Jan group
