# Supplementary material for: Metagenomics of gut microbiome for migratory seagulls in Kunming city revealed the potential public risk to human health
Source: BMC Genomics. 2023 May 19;24:269. doi: 10.1186/s12864-023-09379-1 (PMC10196292; doi:10.1186/s12864-023-09379-1)
Supplement: Supplementary file 6 — Additional file 6. [file 12864_2023_9379_MOESM6_ESM.pdf]

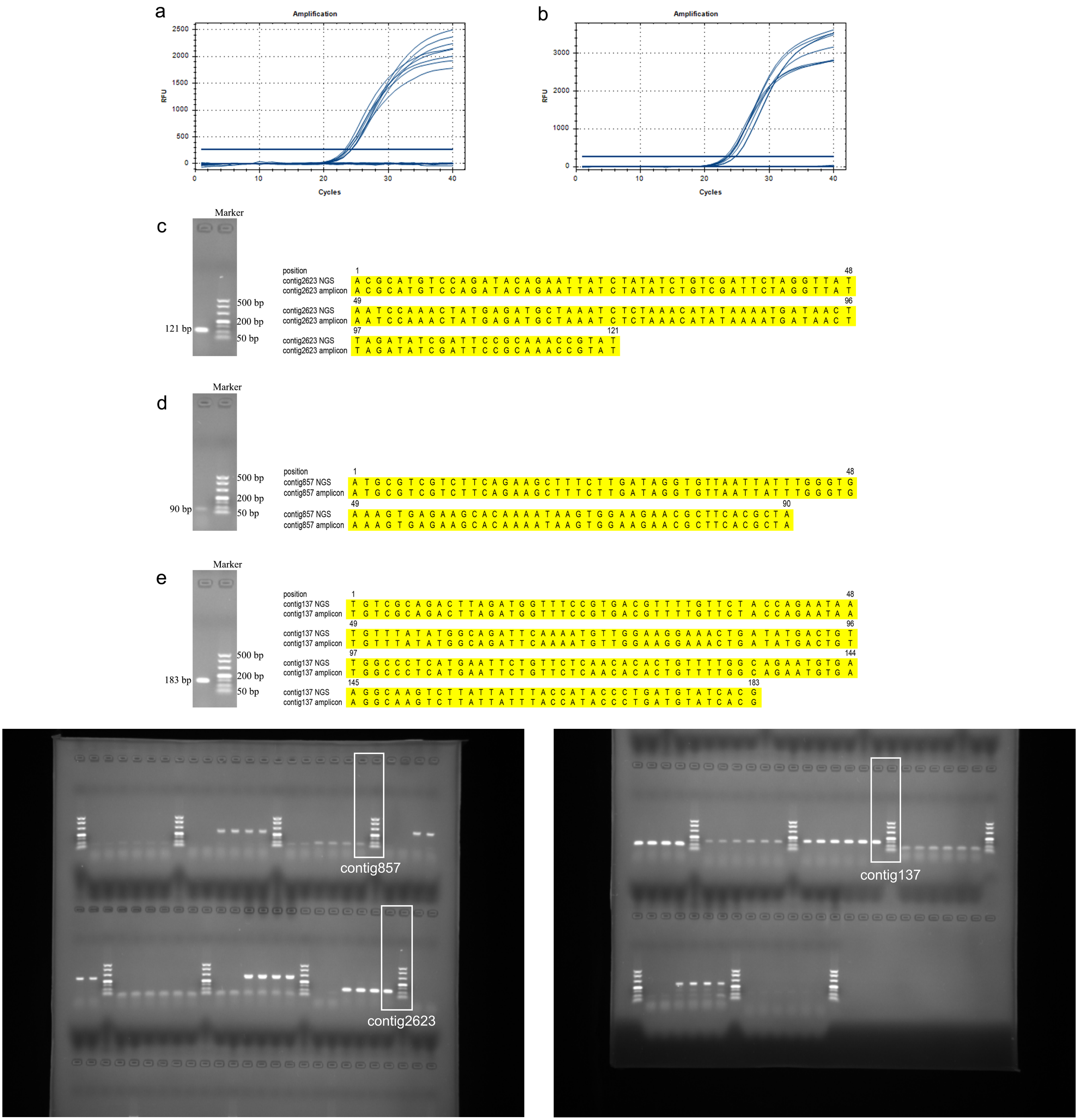

Additional file 6. Verification of NGS data in this study.

a. Real-time PCR results for Salmonella spp. detection. The amplification curves were 8 seagull feces samples, and all the environmental samples were negative

b. Real-time PCR results for Shigella spp. detection. The amplification curves were 8 seagull feces samples, and all the environmental samples were negative

c. The agarose gel electrophoresis and sequence alignment of contig 2623 (The original gel electrophoresis was shown below)

d. The agarose gel electrophoresis and sequence alignment of contig 857 (The original gel electrophoresis was shown below)

e. The agarose gel electrophoresis and sequence alignment of contig 137 (The original gel electrophoresis was shown below)
