# Supplementary material for: Metagenomics of gut microbiome for migratory seagulls in Kunming city revealed the potential public risk to human health
Source: BMC Genomics. 2023 May 19;24:269. doi: 10.1186/s12864-023-09379-1 (PMC10196292; doi:10.1186/s12864-023-09379-1)
Supplement: Supplementary file 7 — Additional file 7. [file 12864_2023_9379_MOESM7_ESM.pdf]

Additional file 7. The primers used for PCR amplification of some novel viruses in RNA virome

Contigs	Forward (5')	Reverse (3')	Lengths	Annealing temp
contig_2623	ACGCATGTCCAGATACAG	ATACGGTTTGCGGAATCG	121 bp	55℃
contig_857	ATGCGTCGTCTTCAGAAGC	TAGCGTGAAGCGTTCTTCC	90 bp	55℃
contig_137	TGTCGCAGACTTAGATGG	CGTGATACATCAGGGTATGG	183 bp	55℃
